# Cadaveric and Ultrasound Validation of Percutaneous Electrolysis Approach at the Distal Biceps Tendon: A Potential Treatment for Biceps Tendinopathy

**DOI:** 10.3390/diagnostics12123051

**Published:** 2022-12-05

**Authors:** Laura Calderón-Díez, José L. Sánchez-Sánchez, Pedro Belón-Pérez, Miguel Robles-García, Fátima Pérez-Robledo, César Fernández-de-las-Peñas

**Affiliations:** 1 Department of Physical Therapy, Universidad de Salamanca, 37008 Salamanca, Spain; 2 Real Madrid C.F., 28055 Madrid, Spain; 3 Department of Anatomy and Histology, Faculty of Medicine, Universidad de Salamanca, 37008 Salamanca, Spain; 4 Department of Physical Therapy, Occupational Therapy, Physical Medicine and Rehabilitation, Universidad Rey Juan Carlos (URJC), 28922 Madrid, Spain; 5 Cátedra Institucional en Docencia, Clínica e Investigación en Fisioterapia: Terapia Manual, Punción Seca y Ejercicio Terapéutico, Universidad Rey Juan Carlos, 28922 Madrid, Spain

**Keywords:** distal biceps brachii tendon, cadaver, brachial artery, tendinopathy, percutaneous electrolysis

## Abstract

Distal biceps brachii tendinopathy is a musculoskeletal pain condition—comprising chronic intrasubstance degeneration with alterations of the tendon structure—that is difficult to treat. Preliminary evidence suggests a positive effect for pain and related disability of percutaneous electrolysis treatment in patients with tendinopathy. Ultrasound is an excellent diagnostic tool to identify tendon injuries, such as tendinopathy, and to guide treatment approaches. Different approaches using ultrasound evaluation of the biceps tendon have been described. Our aim was to determine the validity and safety of a percutaneous electrolysis approach, targeting insertion of the distal tendon of biceps brachii, in both human (ultrasound-guided) and Thiel-embalmed cadaver (not ultrasound-guided) models. There were two approaches evaluated: an anterior approach with the elbow in extension and the forearm in supination and a posterior approach with the elbow in flexion and the forearm in pronation. A needle was inserted following the tendon up to its insertion into the radial tuberosity. The anterior approach, both in cadaveric study and US-guided intervention, revealed a close relationship between the distal biceps tendon and the brachial artery. The mean distance of the depth of the biceps tendon distal to the brachial artery was 0.21 ± 0.021 cm in the cadavers and 0.51 ± 0.024 cm in subjects. It was also found that the anterior approach has a potential technical difficulty due to the anatomical location of the brachial artery. With the posterior approach, it was possible to safely identify the tendon insertion and the needle approach, since no important vascular and nervous structures were visualized in the window of insertion of the needle. The clinician rated the posterior approach as low difficulty in all subjects. Current results would support a posterior approach with US guidance as a safe approach for applying the percutaneous electrolysis technique for insertional tendinopathies of the distal biceps brachii tendon. The current study did not assess the effectiveness of the proposed intervention; accordingly, future studies investigating the clinical effectiveness of the proposed intervention are needed.

## 1. Introduction

Current data support that tendinopathies are the consequence of the response of the failed tendon healing, as well as hypervascularization and alterations of the collagen fibers and the extracellular matrix [1,2]. Distal biceps brachii tendinopathy (BT) is a musculoskeletal pain condition comprising chronic intrasubstance degeneration with alterations of the tendon structure that sometimes evolve or coexist with a partial tear of the tendon at its distal insertion [3]. Injuries affecting the distal biceps tendon are relatively rare in relation to other tendon diseases, but in recent years, a significant prevalence increase has been observed, which is possibly related to an increase in physical activity in older population and improved diagnostic capabilities [4]. BT usually occurs in males between the ages of 40 and 50. It occurs, especially, in manual workers supporting high loads and in some sports, such as weightlifting, where the distal tendon of the biceps brachii is under repetitive mechanical overexertion [5]. Currently, the incidence of BT has been reported to be 1.2 per 100,000 habitants, and it represents 3% of the injuries affecting this region [4]. BT causes pain in the anterior region of the elbow, resulting in significant functional impairment. Patients report gradual onset pain, with or without specific trauma, and develop progressive symptoms such as weakness in elbow flexion with forearm supination [6,7].

The distal tendon of the biceps brachii (DBt) is a flat tendon that begins about 7 cm proximal to the anterior crease of the elbow. It inserts into the bicipital tuberosity of the radius in a double fascicle, occupying an approximate extension of about 21 mm in length and 7 mm in width, which is somewhat less than the total surface of the tuberosity [8]. It has an oblique course from medial to lateral as it deepens toward its insertion on the posterior and ulnar margin of the radial tuberosity. The brachial artery, the main blood vessel of the arm, and the median nerve run very close superficially and/or laterally to the tendon [8].

The anatomical location most vulnerable to injury and degeneration of this tendon is at its insertion, which is approximately 1 cm above the radial tuberosity [9]. There are two main theories explaining DBt injury at this level. The first is related to vascularization since the insertion is a poorly vascularized area that may predispose to tendinopathy at this level. The second theoretical predisposition is related to mechanical impingement of the tendon at the proximal radial-ulnar joint. Cadaver studies have shown a 50% reduction in the space in the proximal part of this joint from maximum supination, so repetitive movements in pronation-supination could result in tendon involvement [5,10].

Ultrasound (US) is an excellent diagnostic tool to identify tendinopathies. It has many advantages—it is less expensive, can be performed dynamically, it has less contraindications—when compared with other diagnostic imaging tests such as Magnetic Resonance Imaging (MRI) [11,12]. Different approaches to US evaluation of the biceps tendon have been described [13]. The anterior approach with the arm extended and the forearm supinated is the most routinely used in clinical practice. However, given the oblique and posterior trajectory of the tendon, US evaluation of the insertion at the radial tuberosity using this anterior approach is often difficult due to the anisotropic effect of the surrounding tissues [13].

A posterior approach with elbow flexion and forearm pronation has been described as an alternative method of US evaluation for examination of the distal tendon insertion in the radial tuberosity [14]. Balius [15] called this approach the “cobra position”, which allows a proper visualization of the insertion of the biceps tendon within the radius. In this position, with the US transducer in the transverse plane, the radial tuberosity faces posteriorly, becoming more superficial, exposing the distal insertion of the biceps tendon. There is no anisotropy, and the fibrillary pattern of the tendon could be better visualized.

Regarding treatment, there is little evidence to guide clinical decisions or effective treatment options to restore function and decrease pain in BT. Optimal treatment remains controversial. It seems reasonable to consider that, in the case of BT, as in other tendinopathies, conservative treatment with physiotherapy, based mainly on tendon load control, would be the best initial option [4]. If symptoms are persistent, other therapies, such as extracorporeal shock wave therapy or platelet-rich plasma [PRP] injections, may also be good treatment options [16,17]. However, the results with these treatments are variable. Current scientific literature consists of case reports and retrospective studies [6], and there is no single treatment considered as the gold standard for the management of BT. In recent years, physical therapy has developed minimally invasive techniques to treat musculoskeletal conditions. Among them, percutaneous electrolysis and dry needling stand out for their effectiveness in the reduction in pain and improvement of function in tendinopathies [18]. Percutaneous electrolysis is an intervention that involves the application of a galvanic electric current through a solid filament needle. There are different studies reporting potential good results of this technique, in terms of pain and function, in different tendinopathies, such as lateral epicondylalgia [19] or supraspinatus tendinopathy [20]. A meta-analysis found moderate evidence suggesting a positive effect of US-guided percutaneous electrolysis for pain and related disability in patients with tendinopathy and musculoskeletal pain [18]. In addition, a secondary analysis has recently revealed that this intervention is cost-effective, at least, for the management of patellar tendinopathy [21]. In addition, a study has observed that percutaneous electrolysis, applied in an animal model of tendinopathy, is able to increase the expression of some genes associated with collagen regeneration and extracellular matrix remodeling, suggesting a potential regenerative effect of tendon tissue of this intervention [22].

To date, there are no studies that have analyzed the effects of the application of this procedure on BT. Due to the anatomical location of the DBt and its surrounding structures, established criteria of uniformity, in terms of methodology, course, and relationship of the DBt, will allow a safe application of invasive US-guided physiotherapy procedures in this area. Accordingly, the aim of this study was to determine the validity and safety of the percutaneous electrolysis approach targeting the insertion of the DBt by using both human (ultrasound-guided) and Thiel-embalmed cadaver (not ultrasound-guided) models. We hypothesized that a posterior approach (“Cobra” position) will permit a better visualization and easier approach of the DBt than an anterior approach.

## 2. Methods

### 2.1. Study Design

A cadaveric and human validation study was conducted. For the cadaveric part, 10 upper extremities from cadavers embalmed in Thiel, donated at the institutional laboratory of the Autonomous University of Madrid (Spain), were used. The specimens were checked for the presence of any structural anomaly that could influence the anatomical status.

### 2.2. Subject Enrollement

There were 10 healthy volunteers who participated in the US-guided part of the study. Participants were recruited by local announcement at the University. Since this was a validation study, we included people without pain symptoms in the upper extremity and without previous neck or upper extremity surgery. The procedure involving healthy participants was performed following the Helsinki Declaration and was approved by the Human Research Ethics Committee (CBE) of the University of Salamanca, Spain (CBE-2021/550). Participants signed a written informed consent form before inclusion. No compensation of any type was provided to the participants.

### 2.3. Anatomical Procedure on Thiel-Embalmed Cadaver

Elbow samples of five specimens (bilateral) were used. The upper extremities were dissected in the long axis, longitudinally, from 10 cm proximal to the elbow to 15 cm distal. The skin and subcutaneous fascia tissue of the dorsal and anterior aspect of the forearm were removed. This allowed for the visualization of the biceps brachii muscle, its distal insertion, the brachial artery and vein, and the median nerve.

A needle was inserted in the cadaver following the tendon up to its insertion in the radial tuberosity, as this is the area where degenerative processes most frequently occur in tendinopathies. All the procedures in which a needle was inserted were performed with a solid filiform needle of 25 × 0.3 mm (AguPunt, Barcelona, Spain).

Two approaches were performed: an anterior approach, with the elbow in extension and forearm in supination, and a posterior approach, with the elbow in and the forearm in pronation. The needle was left in situ during anatomic dissection to determine if the needle tip correctly reached the tendon insertion.

**Figure 1:**
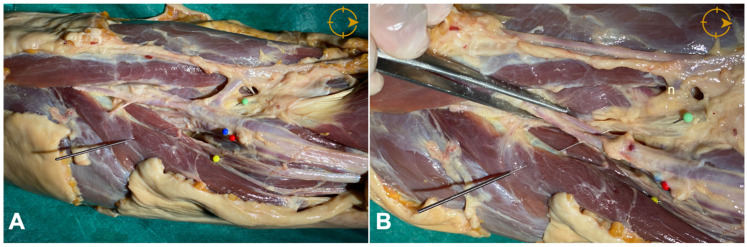
(**A**) Anatomical schematic of the relationship between the brachial artery and vein (blue and red pins), the median nerve (yellow pin), and the distal biceps tendon (green pin). (**B**) Detailed view (orientation arrow indicates: cranial).

**Figure 2:**
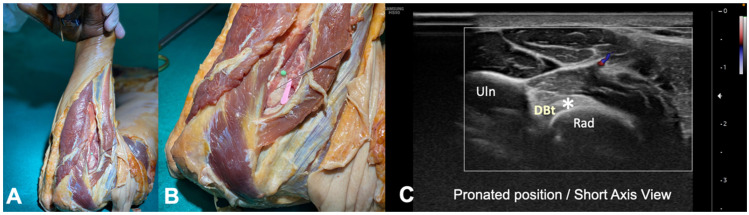
Anatomical schematic of the needle insertion with the elbow in flexion and the forearm in pronation “Cobra position”: (**A**) general view; (**B**) detailed view (tendon insertion-green pin). (**C**) US imaging of the short axis view (pronated position). The tendon insertion (hyperechoic) is observed on the radius tuberosity; (DBt)-Distal Biceps Tendon (Asterisk), (Uln)-Ulna, (Rad)-Radius). No important vascular or nervous structure was visualized (Color Power Doppler image).

In the anterior position, the proximity of the brachial artery was noted. The distance between the upper edge of the biceps tendon and the brachial artery was measured with a digital caliper (Electro DH Mof. 60.220) at 0.5 cm from the insertion (Figure 1). In the posterior approach, the tendon insertion was identified, and it was verified that no important vascular or nervous structures close to the insertion were observed (Figure 2). Cadaveric study was performed without US guidance.

### 2.4. Percutaneous Electrolysis Procedures

The intervention with percutaneous electrolysis was performed in 10 healthy subjects targeting the distal insertion of the biceps tendon. The intervention was US-guided by using a HS-50 Samsung^®^ (Seoul, Republic of Korea), a device equipped with a 14 MHz superficial linear transducer (LA3-14AD). The procedures were performed by a physical therapist with 15 years of experience in musculoskeletal US guided needling interventions. The US depth was set at 3.5 cm to ensure repeatability of the study in all subjects.

**Figure 3:**
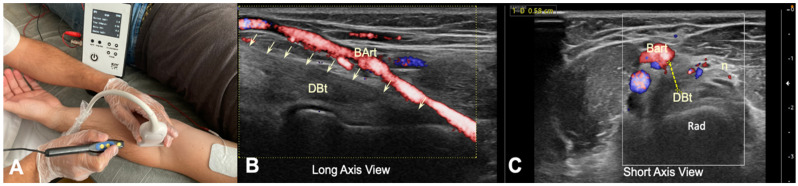
(**A**) Illustration of the percutaneous electrolysis approach with the elbow extended and supinated; (**B**) US imaging (Color Power Doppler) of the relationship between the brachial artery and the distal biceps tendon in long axis, with the elbow extended and supinated. (**C**) US image (Color Power Doppler) in short axis with the forearm supinated of the relationship and measurement of the brachial artery and median nerve to the tendon in a healthy volunteer. (DBt)-Distal Biceps Tendon, (Bart)-Brachial artery, (n)-Median nerve), (Rad)-Radial Tuberosity).

There were two procedures, both US-guided, performed in the same positions as the cadaveric study. In the anterior approach, the DBt was identified, in both long and short axes, as a relatively hyperechogenic structure. The brachial artery and the vein were visualized and identified with the aid of Color Power Doppler. The distance between the center of the superior border of the tendon and the center of the inferior border of the brachial artery was measured ultrasonographically in the short axis. A needle was inserted, with the transducer placed in the long axis of the tendon, until its insertion in the radial tuberosity (Figure 3).

**Figure 4:**
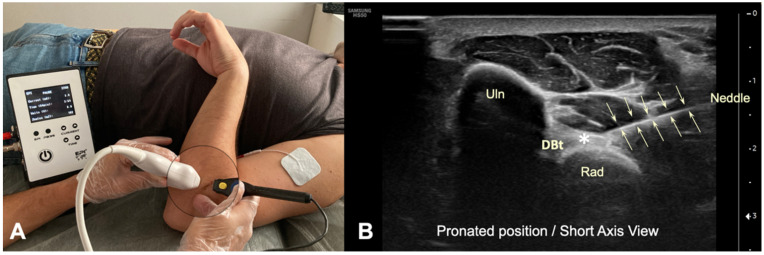
(**A**) Illustration of the percutaneous electrolysis approach with the elbow flexed and pronated in “Cobra position”; (**B**) US imaging of the needle inserting into the distal biceps tendon in short axis, with the elbow flexed and pronated. (DBt)-Distal Biceps Tendon (Asterisk), (Uln)-Ulna, (Rad)-Radius).

In the posterior position, with the US transducer in a transverse position, the tendon insertion was identified. Performing a Color Power Doppler study, neither the brachial artery nor any important vascular or nervous structure was visualized in this case in the planned approach window with the needle (Figure 2C). A needle was inserted to the tendon insertion, in the bicipital tuberosity of the radius, from radial to ulnar (Figure 4).

### 2.5. Statistical Analysis

Statistical analysis was performed with the SPSS statistical package (Version 25.0). Data are presented as means ± standard deviations. Independent Student’s *t*-tests were used to determine differences in brachial artery distance between cadaver samples and healthy subjects. Statistical significance was set at a value of *p* < 0.05.

## 3. Results

This study included 10 healthy volunteers (4 females, mean age: 45 ± 14 years, weight: 78 ± 4.8 kg, height: 175 ± 1 cm, body mass index: 25.0 ± 1.7 kg/cm^2^; 6 males, age 37 ± 12 years, weight: 85.5 ± 5.0 kg; height: 176 ± 3 cm; body mass index: 27.5 ± 2.4 kg/cm^2^) and 10 upper limbs from five cadaver specimens (3 males, mean age: 63 ± 7 years, and 2 females, mean age: 70 ± 11 years).

The anterior approach, both in cadaveric study and in US-guided interventions, has shown that the relationship between the DBt and the brachial artery is close. In all subjects, the artery was observed to run superficial (80%) or superficial and slightly lateral to the biceps tendon (20%). The mean distance of the depth of the DBt to the brachial artery was 0.21 ± 0.021cm in the cadavers studied and 0.51 ± 0.024 cm in the healthy subjects (*p* < 0.01), with both cases at a distance of 0.5 cm from the insertion of the biceps tendon at the tuberosity of the radius (Figure 1 and Figure 3B,C).

The brachial artery was not punctured in any of the needle approaches performed on the volunteer subjects, as the clinician used US guidance. Similarly, no puncture of the brachial artery was visualized on cadavers. However, it was found that the anterior approach has a considerably high technical difficulty, due to the anatomical situation of the brachial artery that interposes between the tip of the needle and the biceps tendon in the long axis US view (Figure 3B). In our series, in 8 of the 10 healthy volunteers (80%), the clinician was forced to move the transducer out of plane when targeting the DBt to avoid inserting the needle into the brachial artery.

Regarding the posterior approach, in the cadaveric study performed with the elbow in flexion and forearm in pronation (“cobra position”), it was possible to identify the tendon insertion, and the needle approach was safe since no vascular and nervous structures were visualized in the window of insertion of the needle (Figure 2C and Figure 4).

The application of percutaneous electrolysis with US was performed correctly, and the clinician rated it as low difficulty in all individuals. Neither the brachial artery nor the median nerve was injured in any of the subjects; therefore, the posterior approach was perceived as a safer approach, performed with US guided control, as compared to the anterior approach. 

## 4. Discussion

The results of this pilot study revealed that the application of US-guided percutaneous electrolysis targeting the DBt insertion, an intervention involving solid puncture with a galvanic current, could be safely performed from a posterior approach. The results also showed that the needle did not puncture any vascular or nervous surrounding tissue.

Ultrasound is an excellent tool for the study and evaluation of tendon pathology, but it is also an essential support in the intervention of some therapeutic procedures with needles. It is an effective and reliable method to minimize the risk of injury [11,12,23]. The lack of clearly identifiable landmarks on palpation, the complex anatomy of the distal biceps with difficulty in visualizing the target area, and the proximity of neurovascular structures (such as the brachial artery and median nerve) are considerable challenges for precise needle placement by US-guided procedures at the DBt insertion. As it has been shown in this study, the brachial artery has a close anatomical relationship with the DBt, and due to its proximity, this structure is at a risk during some procedures on the tendon when using an anterior approach. Iatrogenic injuries of the brachial artery have been described as a complication in needle procedures (such as US-guided infiltrations) on the biceps tendon with an anterior approach [23,24].

Cadaveric studies allow the identification of therapeutic interventions with potential risk for neurovascular tissues that other methods do not allow. In our study, it has been observed that, both in cadaveric models and during US-guided intervention in volunteer subjects, the posterior approach with pronated forearm is easier and safer for applying a needle procedure performed over the distal insertion of the biceps tendon than the anterior approach with supinated forearm since it allows for better visualization of the tendon insertion than the anterior approach, identifying the distal tendon successfully in all the subjects of the study and proving its anatomical correspondence in the cadaveric models.

In the anterior approach, the clinician was forced to move the transducer out-of-plane in order to target the tendon while avoiding inserting the needle into the brachial artery. This fact considerably worsens the visualization of the tendon in a tendon where in-plane US visualization is, in fact, already challenging.

Our conclusion is in line with that reported by Sellon et al. [24], who performed a thorough study with cadaveric models injected with yellow latex at the DBt insertion from different approaches. These authors concluded that the approach to the tendon insertion from the “cobra position” was more reliable, from the technical point of view, than the anterior approach since it had less technical difficulty, and they found no risk of neurovascular injury. Anatomical variations are possible, so the use of US in invasive procedures such as percutaneous electrolysis is essential, as it will allow the identification of structures and a safer application of the technique.

Finally, some limitations of the study must be recognized. First, US imaging and anatomical dissections were performed on a small number of individuals and specimens, respectively. Data on gender differences in needle placement could not be collected. Similarly, anthropometric data of the upper extremities could influence the observed distances. Second, we use, as an anatomical reference point, the distal insertion of the tendon, as it is considered the most common area of degeneration in tendinopathies of this tendon. Consequently, data should be considered for this point addressed. Similarly, our results should be considered in individuals with a normal body mass index. We do not currently know if the same reference points could be used in obese individuals. Third, all needle insertions were performed by an experienced clinician. We do not know the safety and accuracy of this needling procedure when applied by a novice clinician or the reliability of either approach. Finally, it is important to note that the current study did not assess the effectiveness of the proposed intervention and that the same approach could be also used in other procedures, such as plasma rich in platelets (PRP) or hyaluronic acid injections.

## 5. Conclusions

The results from this pilot study support the use of a posterior approach with US guidance as a potential safe approach to apply the percutaneous electrolysis technique or other invasive interventions for insertional tendinopathies of the DBt. It is important to note that the current study did not assess the effectiveness of the proposed intervention.

## Data Availability

All data is presented in the text. Further information is available on proper request.
